# Supplementary material for: Non-invasive laminar inference with MEG: Comparison of methods and source inversion algorithms
Source: Neuroimage. 2018 Feb 15;167:372–83. doi: 10.1016/j.neuroimage.2017.11.068 (PMC5862097; doi:10.1016/j.neuroimage.2017.11.068)
Supplement: Supplementary material [file mmc1.pdf]

Supplementary Figures

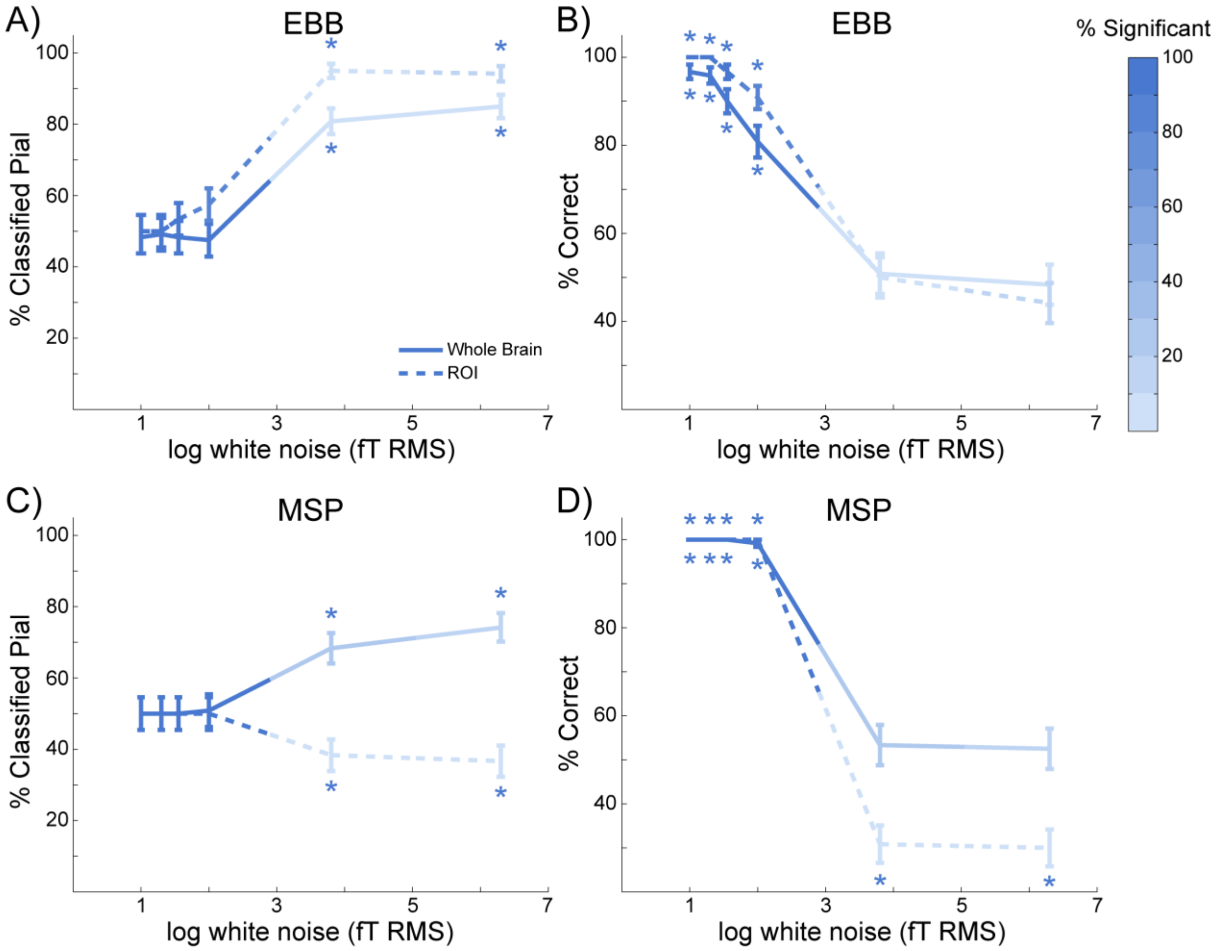

Figure S1: Laminar discrimination accuracy using fixed source magnitudes. *A) The percentage of sources classified as originating from the pial surface for the EBB version of the whole brain and ROI analyses, for each level of white noise tested, with the source signal magnitude fixed at 20nAm. The error bars represent the standard error. The percentage of simulations with free energy differences or t-statistics exceeding the significance threshold is represented by the intensity of the line color. Asterisks show where the percentage is significantly above or below chance levels. Both analyses were unbiased at low noise levels, but became biased towards the pial surface as the noise level increased. B) The percentage of sources accurately classified by the EBB version of the whole brain and ROI analyses, over all tested white noise levels. The classification accuracy of both analyses reached 100% as the white noise level decreased. C) and D) As in (A) and (B) for the MSP version of both analyses.*

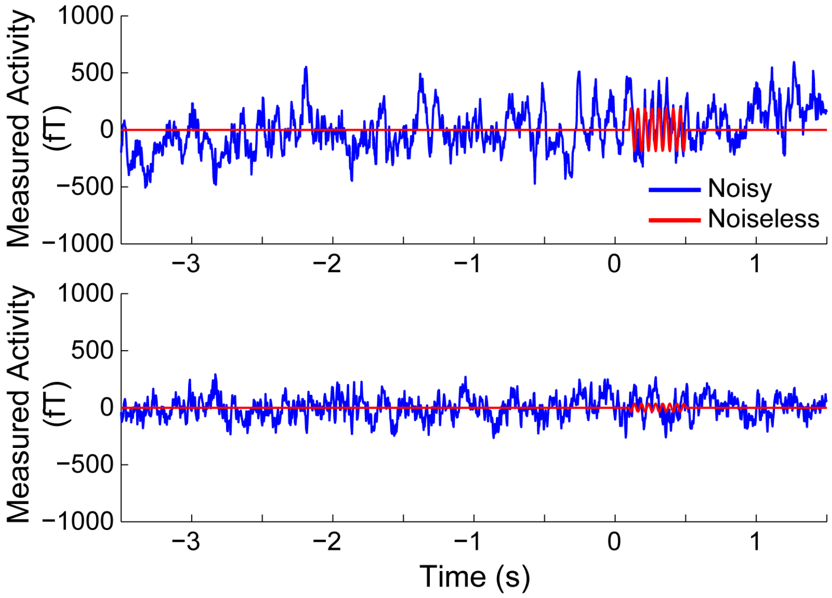

Figure S2: Simulated activity with realistic noise. *Measured data from the sensor with the maximal (top) and median (bottom) signal strength for an example trial. The red traces show the simulated activity without noise (dipole moment = 40nAm) and the blue traces show the resulting activity after adding noise from the resting state data.*

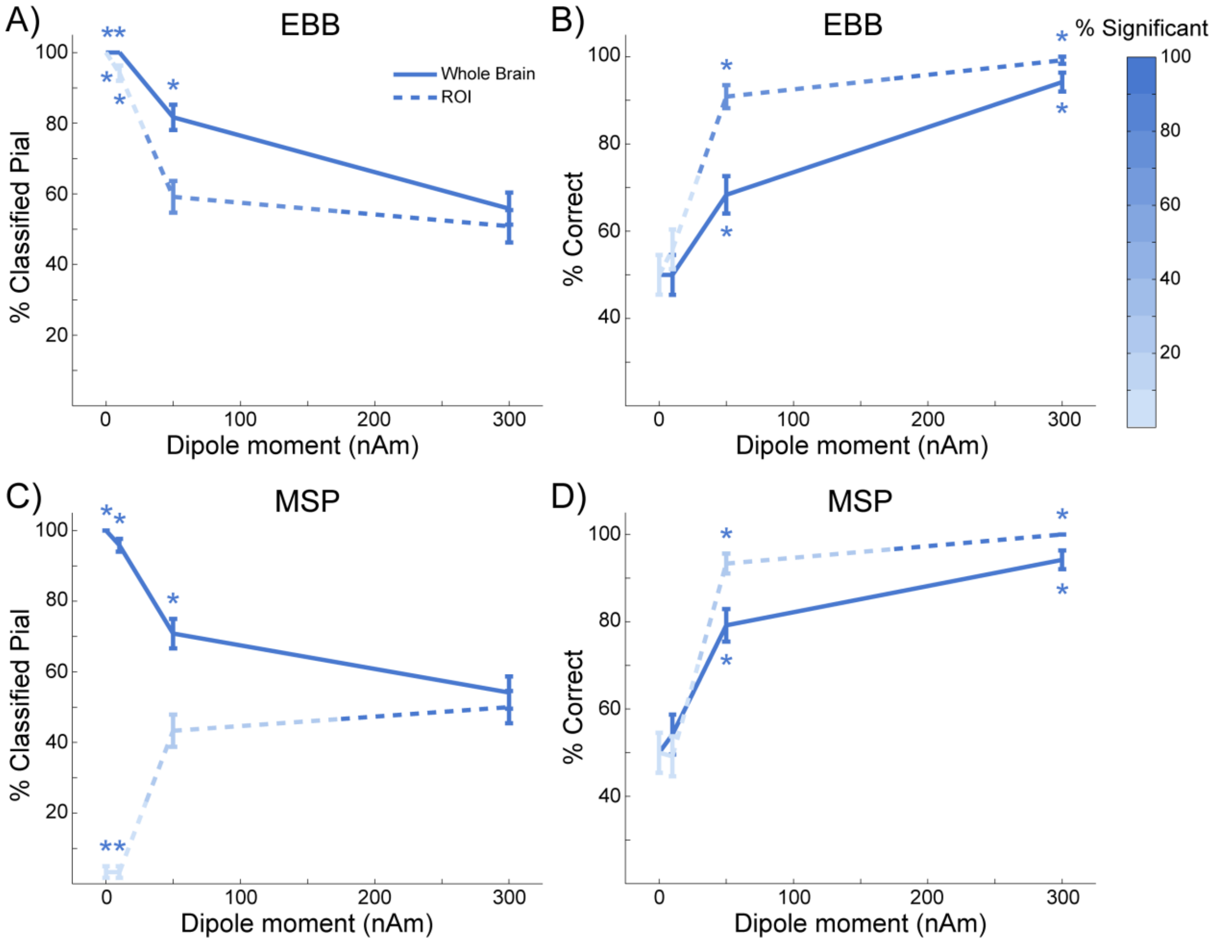

Figure S3: Laminar discrimination accuracy using realistic noise. *A) The percentage of sources classified as originating from the pial surface for the EBB version of the whole brain and ROI analyses, for each level of dipole moment tested, using resting state MEG data as noise. The error bars represent the standard error. The percentage of simulations with free*

energy differences or t-statistics exceeding the significance threshold is represented by the intensity of the line color. Both analyses were biased toward the pial surface at low signal levels, but became unbiased as the signal strength increased. B) The percentage of sources accurately classified by the EBB version of the whole brain and ROI analyses, over all tested dipole moment levels. The classification accuracy of both analyses reached at least 90% as the signal strength increased. C) and D) As in (A) and (B) for the MSP version of both analyses.

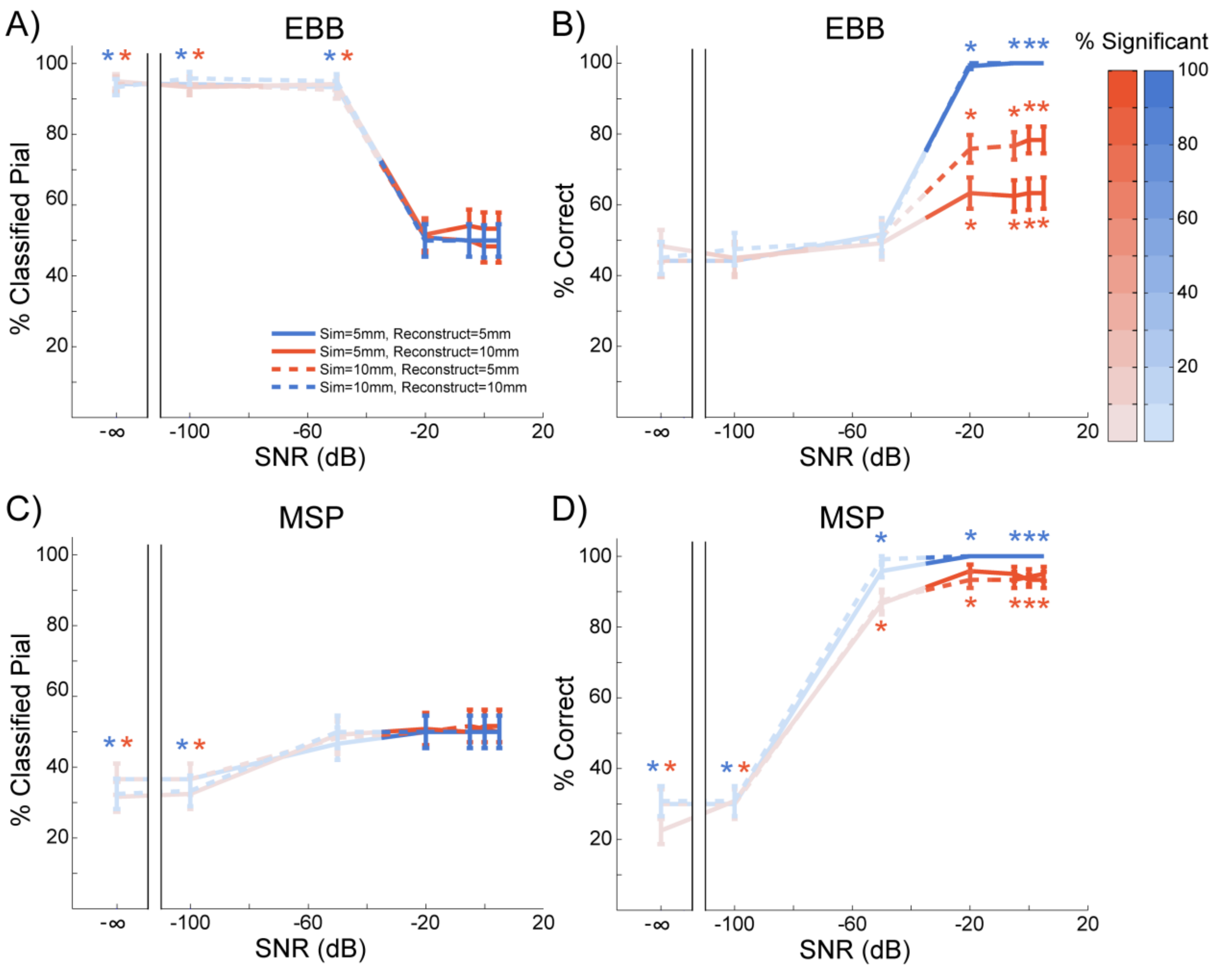

Figure S4: ROI analysis performance with varying patch sizes. *Blue lines denote simulations where the reconstructed patch size matches the simulated patch size (solid=5mm, dashed=10mm), red lines are where patch size is either under- (dashed red) or over-estimated (solid red). The percentage of sources significantly classified as either pial or white matter is represented by the intensity of the line color. Asterisks show where the percentage is significantly above or below chance levels. A) The percentage of sources classified as originating from the pial surface for the EBB version of the ROI analysis, for each level of SNR tested. The error bars represent the standard error. At low SNRs, the algorithm was biased toward the pial surface, but as SNR increased, estimation became unbiased. B) Incorrect patch size estimates resulted in reduced classification accuracy for the EBB version of the ROI analysis. C) The MSP version of the ROI analysis was slightly biased toward the white matter surface, but became unbiased as SNR increased. D) Classification accuracy was reduced for the MSP version of the ROI analysis when the patch size was under- or over-estimated.*

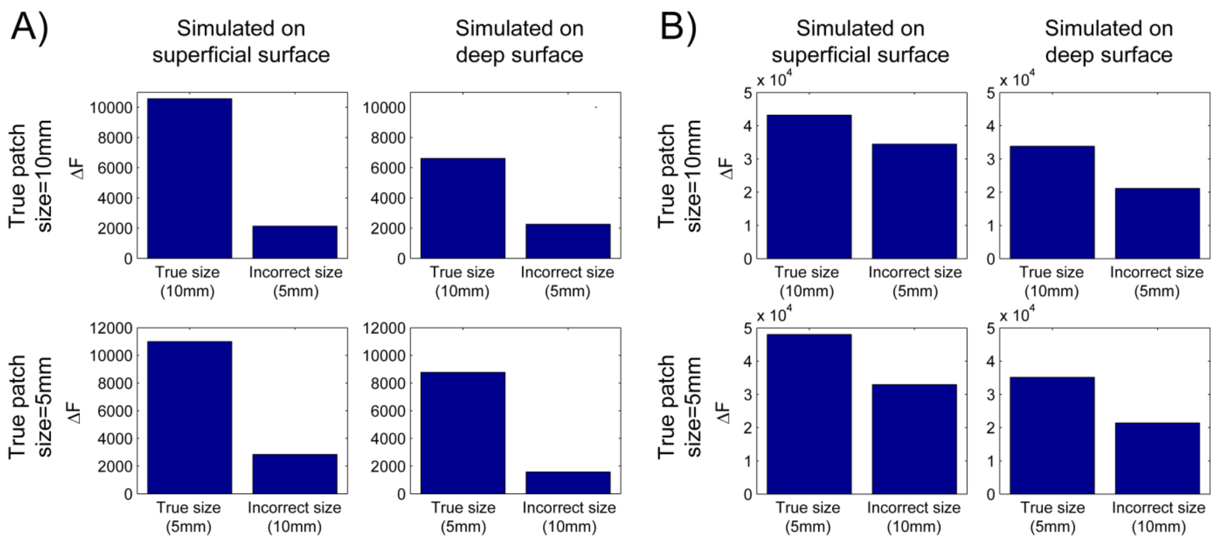

Figure S5: Relative free energy with varying patch sizes. *A) Difference in free energy between the correct and incorrect model (ΔF) using the EBB algorithm with a source simulated on the superficial (left column) or deep surface (right column) with a patch size of 10mm (top row) and 5mm (bottom row) . Each panel shows ΔF with a correct or incorrect estimated patch size. B) As in (A) for the MSP algorithm.*

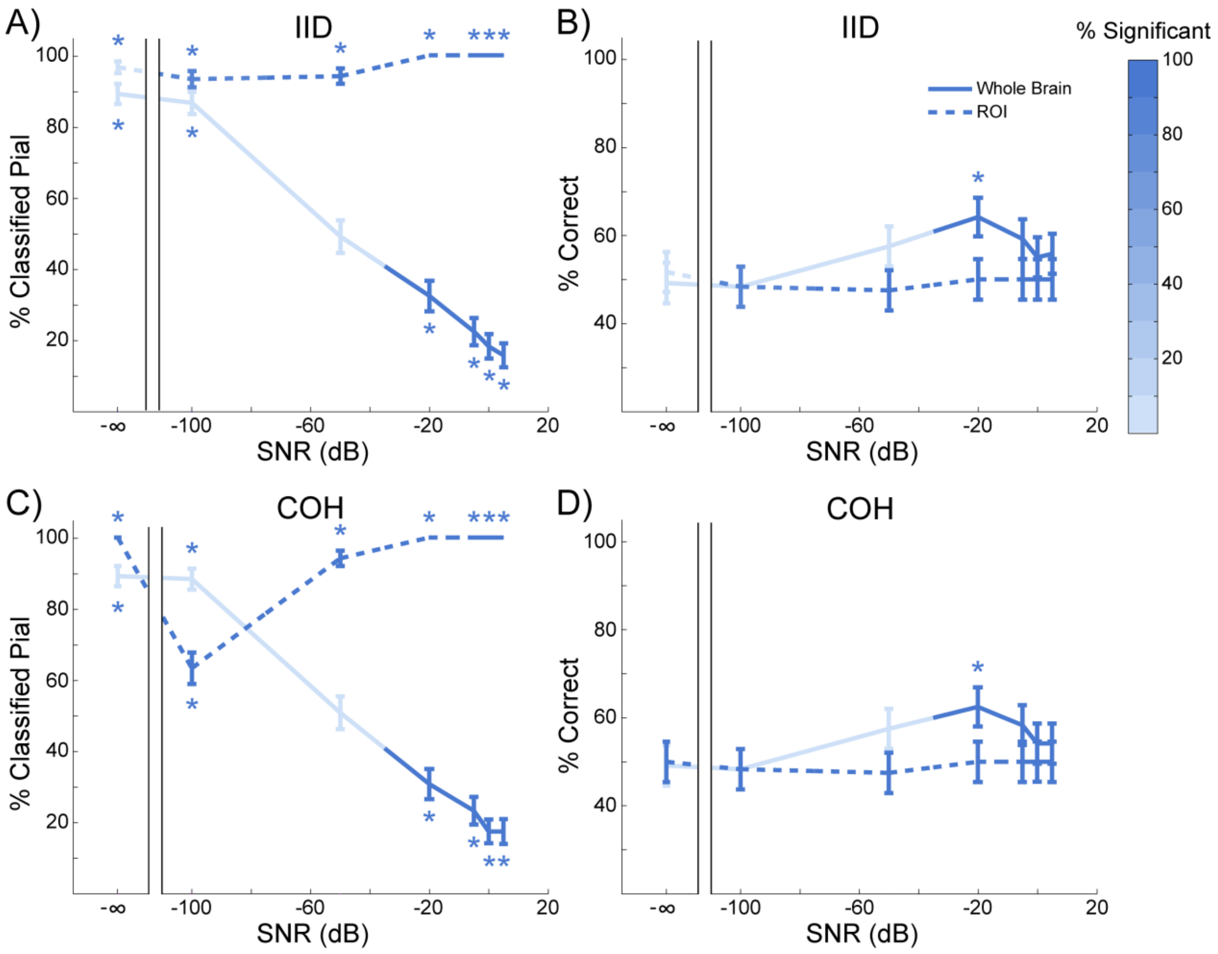

Figure S6: ***Laminar discrimination accuracy of IID and COH algorithms.*** *A) The percentage of sources classified as originating from the pial surface for the IID version of the whole brain and ROI analyses, for each level of SNR tested. The error bars represent the standard error. The percentage of simulations with free energy differences or t-statistics exceeding the significance threshold is represented by the intensity of the line color. Asterisks show where the percentage is*

significantly above or below chance levels. Both analyses were biased toward the pial surface at low SNR levels, but the whole brain analysis becomes biased toward the white matter surface as SNR increases. B) The percentage of sources accurately classified by the IID version of the whole brain and ROI analyses, over all tested SNR levels. Both analyses classified simulated sources at chance levels. C) and D) As in (A) and (B) for the COH version of both analyses. The COH version of the whole brain analysis also became biased toward the white matter surface at high SNR levels and classified sources at chance levels.

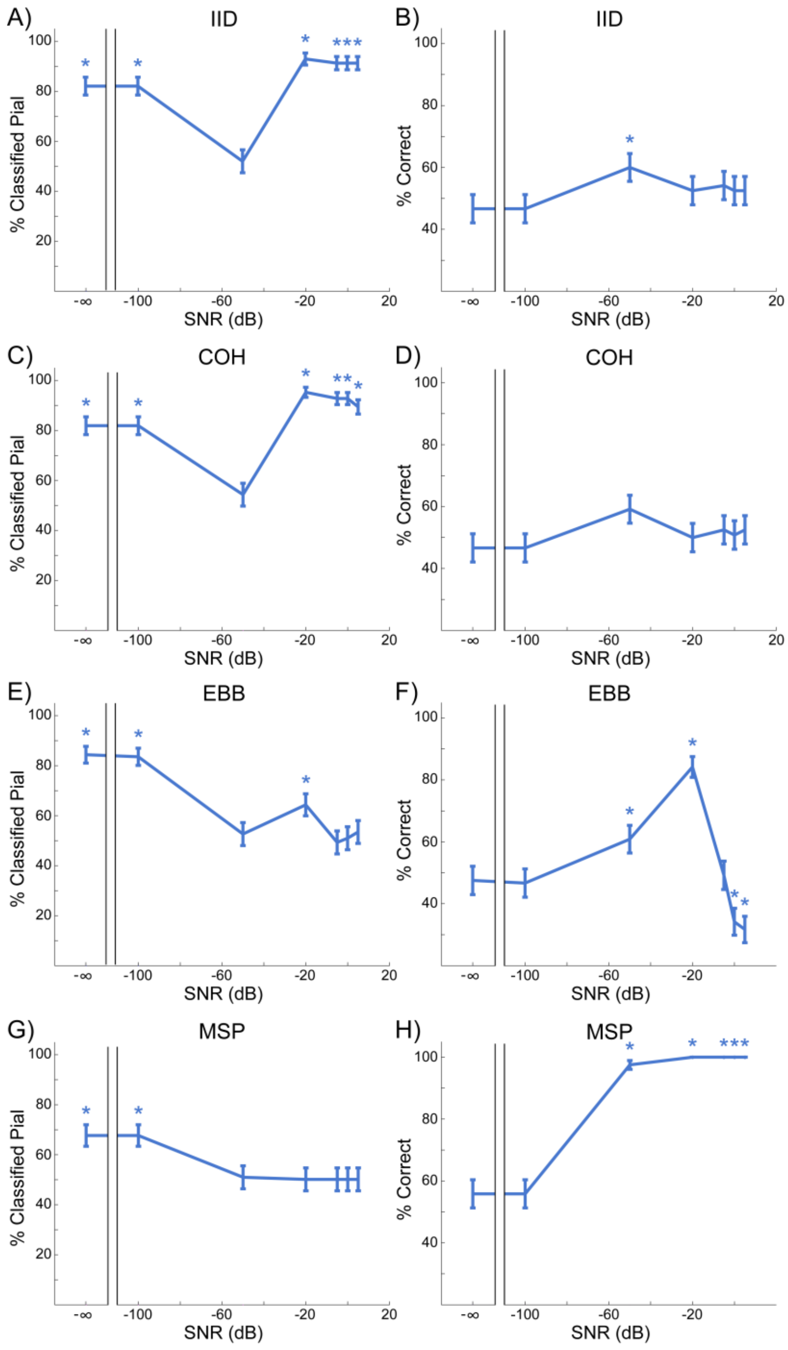

Figure S7: ***Laminar discrimination accuracy using percentage of variance explained.*** *A) The percentage of sources classified as originating from the pial surface for the IID version of the whole brain analysis using the percentage of variance explained as the fit metric rather than free energy, for each level of SNR tested. The error bars represent the standard error. Asterisks show where the percentage is significantly above or below chance levels. The IID algorithm is biased toward the pial surface for all SNR levels. B) The percentage of sources accurately classified by the IID version of the whole brain*

analysis using the percentage of variance explained as the fit metric, over all tested SNR levels. The classification is approximately at chance level for all SNRs. C) and D) As in (A) and (B) for the COH version of the whole brain analysis. Similar to IID, the COH algorithm is biased toward the pial surface and therefore classifies sources at chance levels for all SNRs. E) and F) As in (A) and (B) for the EBB version of the whole brain analysis. The EBB algorithm is biased toward the pial surface at low SNR levels and becomes unbiased as SNR increases. Classification accuracy is at chance levels when SNR is low and increases with SNR up to -20dB and then diminishes as SNR further increases. G) and H) As in (A) and (B) for the MSP version of the whole brain analysis. For the MSP algorithm, the percentage of variance explained yields the same results as the free energy metric. The algorithm is biased toward the pial surface and classifies sources at chance levels when SNR is low, and becomes unbiased and achieves near 100% accuracy as SNR increases.

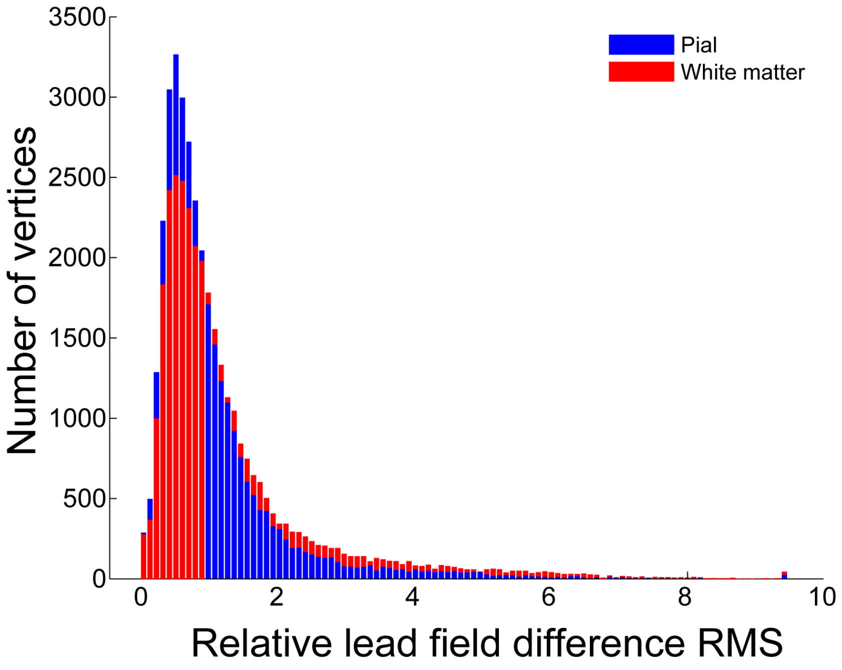

Figure S8: Lead field differences. *Distribution of the ratio of the lead field difference RMS of pial-white matter vertex pairs relative to the lead field difference RMS of neighboring vertices on the pial (blue) and white matter (red) surfaces. Lead fields are more similar between surfaces than within a surface (66.8% of ratios are <1.0 on the pial surface and 56.65% on the white matter surface), but the white matter surface contains more vertices where the lead fields of neighboring vertices are less similar than those of the corresponding pial surface vertices.*
